# Supplementary material for: A Likelihood Approach for Real-Time Calibration of Stochastic Compartmental Epidemic Models
Source: PLoS Comput Biol. 2017 Jan 17;13(1):e1005257. doi: 10.1371/journal.pcbi.1005257 (PMC5240920; doi:10.1371/journal.pcbi.1005257)
Supplement: S1 File — (TAR.GZ) [file pcbi.1005257.s014.tar.gz › HSPH_Online-SI-Revision/IPoi09/n10-severe/n10-severe_BENCH2_table8.pdf]

1	newly 3 weeks spec	53.	118.25	{47., 204.}	128.396	0.277121	newly 3 weeks cumul	226.	441.833	{172., 744.}	104.543	0.248243	newly 5 weeks spec	40.	74.2	{26., 111.}	103.25	0.228956	newly 5 weeks cumul	307.	610.933	{231., 985.}	108.534	0.249119	newly 43 weeks cumul	452.	890.517	{317., 1354.}	110.387	0.239802
2	newly 3 weeks spec	48.	187.54	{84., 363.}	290.708	0.519885	newly 3 weeks cumul	239.	718.619	{349., 1263.}	200.677	0.417179	newly 5 weeks spec	25.	114.46	{49., 229.}	357.841	0.577889	newly 5 weeks cumul	302.	978.444	{469., 1754.}	223.988	0.44647	newly 45 weeks cumul	375.	1361.95	{651., 2409.}	263.187	0.491666
3	newly 3 weeks spec	7.	77.9531	{27., 172.}	1013.62	0.926633	newly 3 weeks cumul	64.	335.75	{135., 751.}	424.609	0.616939	newly 5 weeks spec	1.	40.2344	{11., 84.}	3923.44	1.47093	newly 5 weeks cumul	67.	432.859	{174., 982.}	546.059	0.704992	newly 47 weeks cumul	68.	530.188	{201., 1189.}	679.688	0.78352
4	newly 3 weeks spec	37.	129.655	{43., 213.}	252.47	0.422131	newly 3 weeks cumul	140.	477.466	{183., 850.}	242.278	0.424501	newly 5 weeks spec	17.	86.4138	{25., 142.}	410.953	0.544426	newly 5 weeks cumul	186.	671.483	{239., 1153.}	262.514	0.438593	newly 42 weeks cumul	207.	993.828	{327., 1645.}	380.726	0.534829
5	newly 3 weeks spec	63.	129.655	{43., 213.}	121.128	0.264687	newly 3 weeks cumul	261.	477.466	{183., 850.}	102.358	0.253311	newly 5 weeks spec	22.	86.4138	{25., 142.}	297.492	0.445375	newly 5 weeks cumul	329.	671.483	{239., 1153.}	119.704	0.267422	newly 42 weeks cumul	383.	993.828	{327., 1645.}	168.542	0.312437
6	newly 3 weeks spec	37.	165.197	{68., 297.}	346.478	0.55784	newly 3 weeks cumul	120.	610.727	{260., 1086.}	408.939	0.626541	newly 5 weeks spec	13.	106.955	{41., 185.}	722.727	0.802264	newly 5 weeks cumul	158.	849.545	{352., 1494.}	437.687	0.64411	newly 43 weeks cumul	173.	1228.65	{503., 2188.}	610.203	0.751906
7	newly 3 weeks spec	17.	105.288	{33., 204.}	520.14	0.632873	newly 3 weeks cumul	136.	397.542	{128., 817.}	195.95	0.345495	newly 5 weeks spec	12.	62.9831	{21., 122.}	425.706	0.54066	newly 5 weeks cumul	163.	541.424	{166., 1093.}	234.158	0.384843	newly 42 weeks cumul	196.	757.61	{225., 1460.}	288.404	0.43189
8	newly 3 weeks spec	10.	105.393	{50., 224.}	953.934	0.942928	newly 3 weeks cumul	74.	437.098	{222., 949.}	490.673	0.697143	newly 5 weeks spec	3.	63.0328	{28., 131.}	2001.09	1.22583	newly 5 weeks cumul	80.	581.016	{297., 1296.}	626.27	0.782728	newly 46 weeks cumul	83.	763.344	{395., 1649.}	819.692	0.882809
9	newly 3 weeks spec	28.	98.3548	{46., 170.}	251.267	0.453048	newly 3 weeks cumul	137.	426.081	{209., 668.}	211.008	0.412735	newly 5 weeks spec	9.	48.5161	{21., 85.}	439.068	0.637551	newly 5 weeks cumul	177.	544.516	{257., 857.}	207.636	0.40641	newly 50 weeks cumul	178.	661.081	{304., 1081.}	271.394	0.486922
10	newly 3 weeks spec	9.	37.871	{18., 55.}	320.789	0.555804	newly 3 weeks cumul	56.	186.726	{93., 321.}	233.439	0.45495	newly 5 weeks spec	0.1	17.2742	{8., 33.}	17174.2	2.1491	newly 5 weeks cumul	64.	230.161	{117., 386.}	259.627	0.487915	newly 50 weeks cumul	67.	265.645	{135., 446.}	296.485	0.529886
11	newly 3 weeks spec	0.1	32.3607	{11., 47.}	32260.7	2.36814	newly 3 weeks cumul	7.	158.016	{57., 226.}	2157.38	1.23131	newly 5 weeks spec	0.1	13.4918	{4., 26.}	13391.8	1.98691	newly 5 weeks cumul	7.	192.77	{70., 284.}	2653.86	1.31859	newly 49 weeks cumul	7.	219.016	{76., 325.}	3028.81	1.37363
12	newly 3 weeks spec	18.	91.4516	{35., 149.}	408.065	0.604877	newly 3 weeks cumul	97.	393.5	{161., 624.}	305.67	0.513766	newly 5 weeks spec	11.	45.2097	{15., 73.}	310.997	0.504485	newly 5 weeks cumul	121.	502.516	{198., 785.}	315.303	0.521661	newly 49 weeks cumul	154.	603.887	{231., 968.}	292.134	0.494576
13	newly 3 weeks spec	27.	107.836	{51., 164.}	299.393	0.521679	newly 3 weeks cumul	156.	436.656	{217., 703.}	179.908	0.375498	newly 5 weeks spec	15.	65.	{29., 96.}	333.333	0.540014	newly 5 weeks cumul	190.	585.098	{279., 931.}	207.947	0.414176	newly 46 weeks cumul	207.	787.295	{372., 1239.}	280.336	0.500906
14	newly 3 weeks spec	5.	49.3065	{20., 73.}	886.129	0.880112	newly 3 weeks cumul	40.	217.919	{94., 327.}	444.798	0.639816	newly 5 weeks spec	0.1	22.9032	{9., 38.}	22803.2	2.23072	newly 5 weeks cumul	41.	273.548	{126., 405.}	567.191	0.72517	newly 48 weeks cumul	41.	322.258	{143., 461.}	685.995	0.793938
15	newly 3 weeks spec	2.	12.8889	{5., 24.}	546.032	0.708094	newly 3 weeks cumul	22.	74.8413	{34., 108.}	240.188	0.450997	newly 5 weeks spec	2.	4.69841	{1., 10.}	152.381	0.355063	newly 5 weeks cumul	26.	87.5714	{39., 127.}	236.813	0.447704	newly 52 weeks cumul	28.	94.3333	{41., 134.}	236.905	0.448144
16	newly 3 weeks spec	8.	35.1129	{15., 57.}	338.911	0.544319	newly 3 weeks cumul	58.	153.548	{65., 254.}	165.517	0.335855	newly 5 weeks spec	4.	17.5	{8., 37.}	337.5	0.54141	newly 5 weeks cumul	73.	195.065	{84., 334.}	167.742	0.339517	newly 48 weeks cumul	90.	235.	{97., 413.}	162.366	0.33207
17	newly 3 weeks spec	89.	120.7	{49., 219.}	63.4831	0.195786	newly 3 weeks cumul	412.	491.733	{214., 885.}	54.2557	0.189272	newly 5 weeks spec	62.	68.9833	{27., 119.}	58.1452	0.219923	newly 5 weeks cumul	564.	650.433	{267., 1178.}	54.0426	0.194658	newly 48 weeks cumul	769.	846.6	{344., 1563.}	53.9098	0.203731
18	newly 3 weeks spec	67.	143.689	{73., 249.}	117.054	0.270136	newly 3 weeks cumul	293.	562.951	{282., 920.}	96.0723	0.23476	newly 5 weeks spec	37.	83.4426	{40., 128.}	127.825	0.283768	newly 5 weeks cumul	379.	754.23	{363., 1205.}	102.119	0.243503	newly 46 weeks cumul	562.	1021.43	{485., 1613.}	87.2615	0.210934
19	newly 3 weeks spec	35.	159.121	{55., 284.}	354.631	0.533583	newly 3 weeks cumul	159.	576.81	{217., 1037.}	263.056	0.462725	newly 5 weeks spec	32.	107.603	{35., 166.}	240.894	0.397349	newly 5 weeks cumul	221.	814.069	{295., 1403.}	269.184	0.460916	newly 42 weeks cumul	292.	1222.83	{413., 2054.}	319.816	0.491665
20	newly 3 weeks spec	92.	151.673	{71., 261.}	74.1897	0.209481	newly 3 weeks cumul	401.	554.364	{270., 931.}	51.6663	0.167602	newly 5 weeks spec	59.	106.655	{46., 165.}	90.1387	0.215049	newly 5 weeks cumul	544.	788.291	{369., 1316.}	57.6136	0.176531	newly 42 weeks cumul	745.	1320.25	{543., 1962.}	88.9494	0.20899
21	newly 3 weeks spec	36.	42.1316	{18., 85.}	50.1462	0.19225	newly 3 weeks cumul	108.	197.566	{89., 369.}	90.0463	0.234713	newly 5 weeks spec	14.	18.9079	{7., 39.}	60.6203	0.2061	newly 5 weeks cumul	144.	244.566	{114., 445.}	80.0164	0.217962	newly 50 weeks cumul	181.	283.697	{131., 520.}	72.2666	0.209689
22	newly 3 weeks spec	13.	87.4118	{27., 198.}	572.398	0.683224	newly 3 weeks cumul	94.	388.765	{128., 912.}	313.579	0.481082	newly 5 weeks spec	3.	45.8676	{9., 105.}	1428.92	1.00625	newly 5 weeks cumul	123.	496.853	{162., 1180.}	304.041	0.4662	newly 48 weeks cumul	123.	601.544	{195., 1409.}	389.06	0.54454
23	newly 3 weeks spec	73.	86.1549	{31., 180.}	67.6056	0.237066	newly 3 weeks cumul	250.	355.592	{129., 727.}	74.3268	0.211691	newly 5 weeks spec	30.	47.9718	{17., 103.}	88.7324	0.223593	newly 5 weeks cumul	319.	468.634	{168., 962.}	77.7076	0.212774	newly 47 weeks cumul	375.	601.789	{217., 1229.}	85.7315	0.214173
24	newly 3 weeks spec	15.	98.7213	{45., 176.}	558.142	0.739157	newly 3 weeks cumul	84.	418.787	{188., 752.}	398.556	0.626264	newly 5 weeks spec	11.	53.8361	{22., 84.}	389.419	0.595964	newly 5 weeks cumul	110.	544.23	{252., 957.}	394.754	0.620233	newly 48 weeks cumul	115.	680.934	{314., 1219.}	492.117	0.696125
25	newly 3 weeks spec	0.1	24.3226	{6., 40.}	24222.6	2.23467	newly 3 weeks cumul	2.	127.903	{44., 195.}	6295.16	1.68189	newly 5 weeks spec	0.1	9.43548	{1., 17.}	9338.71	1.7622	newly 5 weeks cumul	2.	152.661	{55., 226.}	7533.06	1.75551	newly 51 weeks cumul	2.	167.29	{56., 253.}	8264.52	1.79348
26	newly 3 weeks spec	24.	35.8955	{11., 79.}	79.5398	0.237794	newly 3 weeks cumul	102.	172.94	{57., 347.}	91.0887	0.245601	newly 5 weeks spec	5.	14.8806	{3., 30.}	210.149	0.399459	newly 5 weeks cumul	124.	210.582	{71., 409.}	91.105	0.243517	newly 50 weeks cumul	128.	237.463	{80., 466.}	103.358	0.258688
27	newly 3 weeks spec	35.	70.6613	{32., 138.}	106.313	0.24965	newly 3 weeks cumul	192.	323.823	{156., 610.}	77.4278	0.198336	newly 5 weeks spec	19.	31.0645	{15., 50.}	77.2496	0.205498	newly 5 weeks cumul	236.	402.145	{190., 750.}	79.0528	0.201471	newly 51 weeks cumul	284.	467.839	{218., 882.}	75.1022	0.195313
28	newly 3 weeks spec	7.	50.2459	{18., 78.}	617.799	0.7354	newly 3 weeks cumul	31.	238.656	{87., 360.}	669.857	0.771026	newly 5 weeks spec	4.	21.2623	{6., 31.}	432.377	0.586482	newly 5 weeks cumul	37.	292.279	{102., 429.}	689.942	0.781236	newly 50 weeks cumul	43.	332.016	{118., 500.}	672.131	0.771135
29	newly 3 weeks spec	0.1	12.1429	{3., 22.}	12042.9	1.93995	newly 3 weeks cumul	12.	68.873	{25., 131.}	473.942	0.647806	newly 5 weeks spec	0.1	3.63492	{0., 8.}	3563.49	1.25308	newly 5 weeks cumul	12.	78.8413	{29., 148.}	557.011	0.703757	newly 52 weeks cumul	12.	83.8571	{30., 153.}	598.81	0.727841
30	newly 3 weeks spec	0.1	26.5085	{10., 46.}	26408.5	2.3437	newly 3 weeks cumul	4.	140.186	{64., 231.}	3404.66	1.47152	newly 5 weeks spec	0.1	11.1017	{4., 20.}	11001.7	1.94332	newly 5 weeks cumul	4.	167.797	{82., 273.}	4094.92	1.55015	newly 52 weeks cumul	4.	186.356	{84., 311.}	4558.9	1.59348
31	newly 3 weeks spec	16.	45.8909	{15., 87.}	189.318	0.311942	newly 3 weeks cumul	78.	218.836	{83., 406.}	183.217	0.322126	newly 5 weeks spec	25.	21.0545	{6., 31.}	70.7636	0.35748	newly 5 weeks cumul	122.	272.818	{107., 498.}	131.401	0.24755	newly 49 weeks cumul	141.	313.691	{121., 581.}	130.11	0.242462
32	newly 3 weeks spec	66.	80.193	{25., 150.}	62.5465	0.222967	newly 3 weeks cumul	192.	314.93	{108., 611.}	85.0603	0.229875	newly 5 weeks spec	25.	47.4035	{13., 87.}	110.246	0.256377	newly 5 weeks cumul	260.	423.842	{146., 815.}	85.135	0.22937	newly 42 weeks cumul	338.	575.228	{183., 1113.}	92.0793	0.23657
33	newly 3 weeks spec	53.	129.655	{43., 213.}	153.221	0.301728	newly 3 weeks cumul	200.	477.466	{183., 850.}	145.922	0.30091	newly 5 weeks spec	33.	86.4138	{25., 142.}	174.608	0.317962	newly 5 weeks cumul	277.	671.483	{239., 1153.}	150.703	0.301558	newly 42 weeks cumul	389.	993.828	{327., 1645.}	165.145	0.308945
34	newly 3 weeks spec	62.	118.25	{47., 204.}	101.156	0.236699	newly 3 weeks cumul	247.	441.833	{172., 744.}	91.6599	0.230078	newly 5 weeks spec	32.	74.2	{26., 111.}	140.417	0.273545	newly 5 weeks cumul	321.	610.933	{231., 985.}	101.651	0.23957	newly 42 weeks cumul	401.	890.517	{317., 1354.}	130.819	0.26602
35	newly 3 weeks spec	19.	49.4154	{18., 78.}	164.939	0.322609	newly 3 weeks cumul	121.	223.338	{78., 325.}	99.021	0.234463	newly 5 weeks spec	16.	23.7077	{6., 40.}	81.25	0.265835	newly 5 weeks cumul	158.	281.877	{108., 427.}	94.2746	0.227212	newly 49 weeks cumul	183.	335.123	{122., 497.}	98.3607	0.233101
36	newly 3 weeks spec	68.	145.365	{68., 292.}	115.406	0.267213	newly 3 weeks cumul	286.	567.413	{271., 1017.}	101.782	0.246769	newly 5 weeks spec	63.	87.7143	{40., 167.}	62.5598	0.191991	newly 5 weeks cumul	404.	769.587	{361., 1413.}	95.2381	0.233412	newly 45 weeks cumul	496.	1065.38	{502., 1901.}	116.996	0.269395
37	newly 3 weeks spec	40.	106.145	{40., 171.}	167.782	0.324547	newly 3 weeks cumul	178.	460.597	{193., 807.}	159.976	0.325196	newly 5 weeks spec	13.	56.2419	{23., 95.}	333.871	0.527814	newly 5 weeks cumul	219.	594.645	{241., 1020.}	172.529	0.341671	newly 48 weeks cumul	249.	734.71	{282., 1193.}	195.738	0.370731
38	newly 3 weeks spec	60.	132.839	{63., 218.}	123.172	0.283234	newly 3 weeks cumul	237.	535.823	{252., 856.}	126.507	0.290135	newly 5 weeks spec	33.	71.5968	{32., 109.}	120.674	0.275531	newly 5 weeks cumul	316.	704.468	{329., 1093.}	123.504	0.282718	newly 48 weeks cumul	375.	903.048	{418., 1396.}	140.951	0.312134
39	newly 3 weeks spec	66.	145.365	{68., 292.}	121.453	0.278123	newly 3 weeks cumul	292.	567.413	{271., 1017.}	98.2224	0.240328	newly 5 weeks spec	58.	87.7143	{40., 167.}	69.2939	0.197642	newly 5 weeks cumul	396.	769.587	{361., 1413.}	98.541	0.23934	newly 44 weeks cumul	641.	1065.38	{502., 1901.}	77.0225	0.201835
40	newly 3 weeks spec	57.	114.46	{49., 229.}	107.602	0.254409	newly 3 weeks cumul	264.	447.365	{205., 851.}	78.7278	0.203084	newly 5 weeks spec	47.	67.9048	{30., 127.}	67.0382	0.197354	newly 5 weeks cumul	347.	602.984	{289., 1130.}	82.2149	0.208518	newly 44 weeks cumul	489.	830.873	{390., 1506.}	80.235	0.205525
41	newly 3 weeks spec	16.	39.75	{14., 59.}	153.854	0.332034	newly 3 weeks cumul	51.	196.217	{76., 293.}	284.739	0.492	newly 5 weeks spec	0.1	16.35	{6., 26.}	16250.	2.10702	newly 5 weeks cumul	55.	237.917	{96., 351.}	332.576	0.543277	newly 52 weeks cumul	59.	264.817	{102., 390.}	348.842	0.558695
42	newly 3 weeks spec	25.	144.933	{51., 231.}	479.733	0.669335	newly 3 weeks cumul	108.	545.217	{211., 929.}	404.83	0.624858	newly 5 weeks spec	2.	94.9	{30., 147.}	4645.	1.54793	newly 5 weeks cumul	125.	758.367	{285., 1280.}	506.693	0.69761	newly 43 weeks cumul	125.	1112.15	{416., 1743.}	789.72	0.837856
43	newly 3 weeks spec	9.	24.95	{10., 36.}	182.037	0.364051	newly 3 weeks cumul	69.	132.483	{60., 250.}	98.1401	0.229701	newly 5 weeks spec	6.	11.5333	{2., 21.}	121.111	0.322382	newly 5 weeks cumul	83.	159.583	{67., 296.}	99.0562	0.231477	newly 51 weeks cumul	83.	176.55	{70., 334.}	118.092	0.267242
44	newly 3 weeks spec	52.	142.73	{52., 308.}	177.961	0.332361	newly 3 weeks cumul	260.	563.063	{221., 1231.}	123.291	0.263432	newly 5 weeks spec	27.	83.3651	{29., 186.}	212.64	0.375957	newly 5 weeks cumul	320.	758.968	{299., 1664.}	141.82	0.286715	newly 45 weeks cumul	421.	1015.29	{372., 2217.}	146.424	0.289444
45	newly 3 weeks spec	23.	79.5738	{37., 112.}	245.973	0.459196	newly 3 weeks cumul	107.	348.475	{174., 486.}	225.678	0.447918	newly 5 weeks spec	11.	41.0492	{18., 60.}	273.174	0.49459	newly 5 weeks cumul	130.	447.23	{224., 624.}	244.023	0.470602	newly 48 weeks cumul	143.	550.59	{284., 784.}	285.028	0.518632
46	newly 3 weeks spec	21.	108.782	{29., 187.}	419.221	0.584148	newly 3 weeks cumul	150.	454.964	{152., 847.}	206.	0.38379	newly 5 weeks spec	19.	56.4545	{15., 96.}	205.167	0.357906	newly 5 weeks cumul	189.	591.836	{192., 1094.}	215.604	0.39034	newly 47 weeks cumul	195.	733.691	{229., 1408.}	277.128	0.455022
47	newly 3 weeks spec	36.	106.274	{43., 171.}	196.013	0.363971	newly 3 weeks cumul	193.	462.21	{198., 807.}	141.543	0.297489	newly 5 weeks spec	17.	56.4032	{23., 95.}	235.389	0.426865	newly 5 weeks cumul	233.	596.774	{243., 1020.}	157.566	0.32031	newly 48 weeks cumul	246.	737.242	{296., 1193.}	200.295	0.378876
48	newly 3 weeks spec	58.	187.54	{84., 363.}	223.344	0.437698	newly 3 weeks cumul	260.	718.619	{349., 1263.}	176.392	0.380604	newly 5 weeks spec	33.	114.46	{49., 229.}	247.042	0.458177	newly 5 weeks cumul	358.	978.444	{469., 1754.}	173.309	0.372594	newly 45 weeks cumul	432.	1361.95	{651., 2409.}	215.267	0.430214
49	newly 3 weeks spec	33.	93.9048	{38., 161.}	186.388	0.356071	newly 3 weeks cumul	141.	394.508	{166., 737.}	181.099	0.353582	newly 5 weeks spec	29.	47.746	{15., 77.}	88.5057	0.241343	newly 5 weeks cumul	202.	511.429	{216., 950.}	156.058	0.316156	newly 47 weeks cumul	230.	635.016	{256., 1188.}	177.902	0.34491
50	newly 3 weeks spec	0.1	12.1803	{4., 19.}	12080.3	1.99662	newly 3 weeks cumul	33.	68.0164	{34., 103.}	109.091	0.259385	newly 5 weeks spec	0.1	4.11475	{1., 8.}	4027.87	1.43836	newly 5 weeks cumul	33.	79.8689	{40., 124.}	143.02	0.320373	newly 52 weeks cumul	33.	86.5902	{42., 136.}	162.494	0.351953
